# New cytogenetic data for three species of Pentatomidae (Heteroptera): *Dichelops melacanthus* (Dallas, 1851), *Loxa viridis* (Palisot de Beauvois, 1805), and *Edessa collaris* (Dallas, 1851)

**DOI:** 10.3897/CompCytogen.v14i4.56743

**Published:** 2020-11-17

**Authors:** Jaqueline Fernanda Dionisio, Joana Neres da Cruz Baldissera, Angélica Nunes Tiepo, José Antônio Marin Fernandes, Daniel Ricardo Sosa-Gómez, Renata da Rosa

**Affiliations:** 1 Universidade Estadual de Londrina (UEL), Rodovia Celso Garcia Cid, PR 445, Km 380, Caixa Postal 10.011, 86057-970, Londrina, PR, Brazil Universidade Estadual de Londrina Londrina Brazil; 2 Instituto de Ciências Biológicas, Universidade Federal do Pará, Belém, Universidade Federal do Pará, 66075-110; PA, Brazil Universidade Federal do Pará Pará Brazil; 3 Empresa Brasileira de Pesquisa Agropecuária/Centro Nacional de Pesquisa de Soja (EMBRAPA/CNPSO), Rodovia Carlos João Strass, 86001-970, Distrito de Warta, Londrina, PR, Brazil Centro Nacional de Pesquisa de Soja Londrina Brazil

**Keywords:** Heterochromatin, Holocentric chromosome, Meiosis, Pentatomidae, rDNA-FISH

## Abstract

In this paper, we present new cytogenetic data for three species of the family Pentatomidae: *Dichelops melacanthus* (Dallas, 1851), *Loxa viridis* (Palisot de Beauvois, 1805), and *Edessa collaris* (Dallas, 1851). All studied species presented holocentric chromosomes and inverted meiosis for the sex chromosomes. *D. melacanthus* has 2*n* = 12 (10A + XY); *L. viridis* showed 2*n* = 14 (12A + XY); and *E. collaris* showed 2*n* = 14 (12A + XY). C-banding was performed for the first time in these species and revealed terminal and interstitial heterochromatic regions on the autosomes; DAPI/CMA_3_ staining showed different fluorescent patterns. In all species, fluorescence *in situ* hybridization (FISH) with 18S rDNA probe identified signals on one autosomal bivalent, this being the first report of FISH application in the species *D. melacanthus* and *L. viridis*. The results obtained add to those already existing in the literature, enabling a better understanding of the meiotic behavior of these insects.

## Introduction

The suborder Heteroptera has approximately 40,000 species distributed in seven infraorders (Enicocephalomorpha, Dipsocoromorpha, Gerromorpha, Nepomorpha, Leptopodomorpha, Cimicomorpha, and Pentatomomorpha) and is considered the largest and most diverse group of hemimetabolous insects (Štys and Kerzhner 1975; Weirauch and Schuh 2011). Although many of these insects play an important role as indicators of environmental quality (Brown 1997), other species are responsible for significant economic importance as vectors of diseases (Alevi et al. 2015) and agricultural pests (Schaefer and Panizzi 2000).

Pentatomidae are considered the fourth largest family in the suborder Heteroptera, with approximately 900 genera and almost 4,800 species classified in 10 subfamilies with a worldwide distribution (Rider 2011). The Neotropical region, which includes Brazil, is known for its vast biodiversity (Goldani 2012), where 230 genera and more than 1,400 Pentatomidae species have already been described (Grazia et al. 2015).

Several cytogenetic studies have been conducted on this insect family, where more than 300 species have been analyzed (Ueshima 1979; Rebagliati et al 2005; Kaur and Semahagn 2010; Kerisew 2012; Souza et al. 2007, 2011; Grozeva et al. 2011; Souza and Itoyama 2011; Bardella et al. 2013a, 2016a). The diploid numbers ranging from 2*n* = 6 in *Rhytidolomia senilis* (Say, 1832) to 2*n* = 27 in *Thyanta calceata* (Say, 1832) with a predominance of 2*n* = 14 and sex chromosome system XX/XY. These insects like the rest species of Heteroptera have specific cytogenetic features, such as (i) holocentric chromosomes; (ii) kinetic activity located in the terminal region of chromosomes during meiosis (telokinetic activity); (iii) chiasmatic autosomal bivalents, in contrast to the sex chromosomes that are achiasmatic; and (iv) inverted meiosis in the sex chromosomes, which is different from the typical equation reduction-sequence observed in the meiosis of organisms with monocentric chromosomes (Ueshima 1979; Nokkala and Nokkala 1984, 1997; Nokkala and Grozeva 2000; Pérez et al. 2000; Papeschi et al. 2003; Poggio et al. 2009; Melters et al. 2012).

Because of the importance and diversity of the family Pentatomidae, we present cytogenetic data for three species of Pentatomidae in this paper: *Dichelops melacanthus* (Dallas, 1851), *Loxa viridis* (Palisot de Beauvois, 1805), and *Edessa collaris* (Dallas, 1851).

## Methods

### Chromosomal preparations and conventional staining

For this study, only male adults were used (Table 1). Specimens were collected with the authorization of the ICMBio (31946-4). The insects were anesthetized and dissected in a physiological solution for insects (7.5 g NaCl, 2.38 g Na_2_HPO_4_, and 2.72 g KH_2_PO_4_ in 1 l of distilled water). The gonads were washed with tap water and fixed in methanol and acetic acid (3:1, v:v). The slides were prepared based on the protocol of Pijnacker and Ferwerda (1984), using a portion of the testes, which was macerated in 45% acetic acid and then dried on a hot plate at 45–50 °C. These preparations were stained using conventional staining with Giemsa 3%.

**Table 1.** Studied species and collection sites.

Species	Number of samples (N)	Collection site
*Dichelops melacanthus*	40	District of Maravilha, Londrina, Paraná, Brazil (23°28'03"S, 51°00'46.3"W)
*Loxa viridis*	15	Iguaçu National Park in Foz do Iguaçu, Paraná, Brazil (25°04'–25°41"S, 53°58'–25°04"W)
*Edessa collaris*	15	Iguaçu National Park in Foz do Iguaçu, Paraná, Brazil (25°04'–25°41"S, 53°58'–25°04"W)

### C-banding and fluorochromes

The slides were submitted to C-banding following the protocol of Sumner (1972) with the modifications of Grozeva et al. (2011). The slides were treated with 0.2 N HCl solution at room temperature for 30 min, incubated in 5% barium hydroxide solution at room temperature for 8 min, and then incubated in 2 × SSC saline at 60 °C for 1 h. The slides were washed with distilled water; some were stained with propidium iodide according to Lui et al. (2012), and others were stained with the fluorochromes 4’6-diamidino-2-phenylindole (DAPI), which identify AT-rich regions, and chromomycin A3 (CMA_3_), which identify GC-rich regions (Schweizer 1980).

### DNA extraction and isolation of the 18S rDNA probe

Total DNA was extracted using the phenol-chloroform method of Sambrook and Russel (2006). The 18S rDNA probe was obtained via a polymerase chain reaction (PCR) using the primers Forward 5'-CCTGAGAAACGGCTACCACATC-3' and Reverse 5'-GAGTCTCGTTCGTTATCGGA-3', as described by Whting et al. (1997). The PCR was performed with a final volume of 25 μl containing 100 ng of genomic DNA (1 μl), 10 mM primer (1 μl each), 10 mM dNTP mix (1 μl), 50 mM MgCl_2_ (1.5 μl), and 10 × PCR buffer (2.5 μl); Taq polymerase at 5 U/μl (0.5 μl) was added to ultra-pure water to complete the reaction. The PCR was used in the following conditions: first step at 94 °C for 2 min, followed by 35 cycles at 94 °C for 1 min, 58 °C for 1 min, 72 °C for 1 min, and a final extension of 72 °C for 5 min. Amplified 18S rDNA probes were labeled using digoxigenin-11-dUTP (Roche Applied Science, Indianapolis, EUA).

### Fluorescence *in situ* hybridization (FISH)

FISH was based on the protocol of Schwarzacher and Heslop-Harrison (2000): the slides were treated with RNase (0.4% RNAse/2 × SSC) and pepsin (0.005%) for 1 h and 10 min, respectively, both at 37 °C, and dehydrated in ethanol series (75% and 100%) for 3 min each. Subsequently, 40 μl of the hybridization mix containing 100% formamide (20 μl), 50% polyethylene glycol (8 μl), 20 × SSC (4 μl), 10% sodium dodecyl sulfate (SDS) (1 μl), and 100 ng of probes (4 μl) was added to ultra-pure water to complete the reaction. The hybridization mix was denatured at 75 °C for 10 min and then was transferred to the ice. After this time, the mix was applied onto the slides and taken to the thermocycler for denaturing/renaturing following the steps (90 °C, 56 °C, and 38 °C for 10 min each); hybridization occurred at 37 °C in a humidified chamber overnight. After probe detection with anti-digoxigenin-rhodamine (Roche Applied Science, Indianapolis, EUA), the chromosomes were counterstained with DAPI and mounted in Vectashield (Thermo Fisher Scientific, Massachusetts, EUA) and left in the dark for 24 h before analysis.

The slides were analyzed in an epifluorescence microscope (Leica DM 2000), which was equipped with a digital camera Moticam Pro 282B. The images were captured using Motic Images Advanced software, version 3.2. The chromosome images were acquired separately with specific filters for each fluorophore or in light field.

## Results

**Figure 1.**
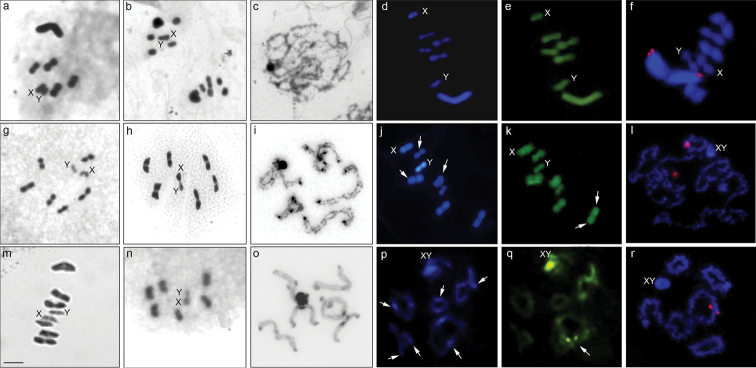
Meiotic stages of *Dichelops melacanthus* (**a–f**), *Loxa viridis* (**g–l**) and *Edessa collaris* (**m–r**). (**a, g** and **m**) metaphase I by Giemsa conventional staining; (**b, h** and **n**) metaphase II by Giemsa conventional staining; (**c, i** and **o**) C-banding pachytenes; (**d** and **j**) metaphase I by DAPI staining; (**p**) diplotene by DAPI staining; (**e** and **k**) metaphase I by CMA_3_ staining; (**q**) diplotene by CMA_3_ staining; (**f, l** and **r**) Fluorescence *in situ* hybridization with digoxigenin-labeled 18S rDNA probe and counterstained with DAPI. X and Y correspond to the sex chromosomes. Arrows show heterochromatic marks in autosomes. Scale bar: 10 µm.

The stink bug *D. melacanthus* had 2*n* = 12 (10A + XY) (Fig. 1a, b) with one pair of autosomes larger than the other autosomes. *L. viridis* (Fig. 1g, h) and *E. collaris* (Fig. 1m, n) had chromosomes of homogeneous size and 2*n* = 14 (12A + XY), in both species. The sex chromosome system of all species was simple (XY male). The analysis of the meiotic behavior revealed a kinetic activity in the terminal regions of the chromosomes owing to their positioning and migration to the opposite poles. In metaphase II, it was possible to observe a radial plate the autosomes forming a ring and the sex chromosomes positioned in the center of the ring in a brief association, known as touch-and-go-pairing in all species (Fig. 1b, h, n).

In all analyzed species, a heterochromatic region corresponding to sex chromosomes was observed, which are associated in the early stages of meiosis (Fig. 1c, i, o). In *D. melacanthus*, the DAPI/CMA_3_ staining was homogeneous for all chromosomes (Fig. 1d, e). In *L. viridis* presented interstitial and terminal heterochromatic regions in some autosomes (Fig. 1i), and after staining with fluorochromes, the Y chromosome stood out as DAPI^+^ (Fig. 1j). A similar pattern was observed in one of the autosomes but with more discrete coloration; the DAPI^+^ interstitial regions were not so evident in another autosome (Fig. 1j). Staining with CMA_3_ showed very weak terminal dots in a bivalent (Fig. 1k). The specie *E. collaris* showed terminal and interstitial heterochromatic bands in the autosomal bivalents in addition to the sex chromosomes that were associated (Fig. 1o). The DAPI/CMA_3_ staining revealed the presence of several interstitial and a terminal DAPI^+^ bands (Fig. 1p) as well as CMA_3_^+^ bright terminal dots in a bivalent, and the DAPI^+^/CMA_3_^+^ sex chromosomes (Fig. 1p, q).

FISH revealed the following distribution patterns of 18S rDNA among species: *D. melacanthus* showed discrete dots in the terminal region of the larger bivalent at metaphase I (Fig. 1f); two 18S rDNA sites in the terminal region of a bivalent were observed in the initial meiotic phases of *L. viridis* and *E. collaris* (Fig. 1l, r).

## Discussion

Conventional staining analysis performed here confirmed the presence of holocentric chromosomes and kinetic activity localized in the terminal region during meiosis, as observed in most Heteroptera (Ueshima 1979; Nokkala and Nokkala 1984, 1997; Nokkala and Grozeva 2000; Pérez et al. 2000; Papeschi et al. 2003; Poggio et al. 2009; Melters et al. 2012). In addition, it was possible to observe the occurrence of inverted meiosis for the sex chromosomes, as evidenced by the presence of these chromosomes as univalents in metaphase I and the presence of heteromorphic chromatids with touch-and-go-pairing behavior in metaphase II, a feature already reported in other species of Pentatomidae (Viera et al. 2009).

According to data available for Pentatomidae (Rebagliati et al. 2005; Bardella et al. 2013a), a diploid number conservation of *L. viridis* (2*n* = 14) was observed. *D. melacanthus* presented 2*n* = 12 (10A + XY), as previously observed by other authors (Rebagliati et al. 2002, 2005; Souza et al. 2011). The population of *E. collaris* analyzed in this study had 2*n* = 14 (12A + XY); however, Souza et al. (2011) reported 2*n* = 12 (10A + XY) for the same species, which may indicate an interpopulation polymorphism because the collection sites were distinct. Another explanation of the difference in the chromosome number of *E. collaris* could be an error in species identification because of the morphological similarity between species in this genus, making identification difficult (Fernandes and Doesburg 2000).

The stink bug *D. melacanthus* was the only species in the study that presented 2*n* = 12 (10A + XY), a result confirming previous observations reported for other populations of this species (Rebagliati et al. 2002, 2005; Souza et al. 2011). This diploid number is described in nine other Pentatomidae species: *Euschistus crassus* Dallas, 1851 (Foot and Strobell 1912 according to Ueshima 1979, Hughes-Schrader and Schrader 1956); *Oechalia patruelis* Stål, 1859 (Heizer 1950), *Scotinophara* sp. (Jande 1959 and 1960c according to Rebagliati et al. 2005), *Scotinophara coarctata* Fabricius, 1979 (Satapathy et al. 1990 according to Rebagliati et al. 2005), *Dichelops furcatus* Fabricius, 1775 (Rebagliati et al. 2001), *Mecocephala maldonadensis* Schwertner, Grazia, and Fernandes, 2002 (Rebagliati et al. 2005), *Acledra bonariensis* Stål, 1859 (Rebagliati and Mola 2010), *Edessa collaris* (Souza et al. 2011) and *Cahara confusa* Distant, 1879 (Kaur and Sharma 2015).

Apart from the differences in the diploid number, *D. melacanthus* was distinguished by the presence of a pair of autosomal chromosomes of large size in relation to the other chromosomes. According to Souza et al. (2011), the presence of this visibly larger autosomal pair suggests that this reduced karyotype originated through a fusion between two autosomes. The same characteristic has also been observed in other species of the family with 2*n* = 12, as in *E. crassus*, *D. furcatus*, *M. maldonadensis*, and *A. bonariensis* (Hughes-Schrader and Schrader 1956; Ueshima 1979; Rebagliati et al. 2001, 2005; Rebagliati and Mola 2010), which supports the hypothesis of the fusions resulting in a reduction of the diploid number in this group.

In all species studied, the location and composition of heterochromatin was first performed. In relation to the characteristics of heterochromatin in the autosomes, we can classify the species studied into two distinct patterns: (i) presence of AT-rich heterochromatin as in *D. melacanthus* and (ii) predominance of DAPI^+^ blocks and few CMA_3_^+^ blocks as in *L. viridis* and *E. collaris*. According to Poggio et al. (2011), most of the reports concerning the characterization of heterochromatin in the autosomes in species of the order Hemiptera are described as DAPI^+^, as was reported by Bressa et al. (2005) in *Athaumastus haematicus* (Stål, 1860), *Leptoglossus impictus* (Stål, 1859), *Phthia picta* (Drury, 1770) (Coreidae), *Largus rufipennis* (Laporte, 1832) (Largidae) and *Jadera sanguinolenta* (Fabricius, 1775) (Rhopalidae), and by Franco et al. (2006) in *Spartocera batatas* (Fabricius, 1798) (Coreidae).

The heterogeneity of heterochromatin in chromosomes was observed. In *D. melacanthus* and *L. viridis*, the sex Y chromosome was completely heterochromatic and DAPI^+^, while the sex X chromosome in these two species showed homogeneous staining with both DAPI and CMA_3_. In most species of Heteroptera, the Y chromosome presents a large amount of heterochromatin, sometimes being completely heterochromatic (Grozeva and Nokkala 2001). This has been reported for the subfamily Triatominae (Panzera et al. 1995), in species of Belostomatidae (Papeschi 1988) and in three species of pentatomids of the genus *Antiteuchus* (Dallas, 1851) (Lanzone and Souza 2006). Although studies in Reduviidae show that the DAPI positive Y chromosome is quite common, particularly in species of the Triatomini tribe (Bardella et al. 2016b), it is not observed with the same frequency in Pentatomidae, being pointed out only in *Halys serrigera* (Westwood, 1837) and *Perillus bioculatus* (Fabricius, 1775) (Kerisew 2012).

In this study, *E. collaris* presented associated sex chromosomes and DAPI^+^/CMA_3_^+^ in early meiotic phases. This has also been reported in *Nabis viridulus* Spinola, 1837 (Grozeva et al. 2004) and in species of the genus *Edessa* in *E. meditabunda* (Fabricius, 1974) *and E. rufomarginata* (De Geer, 1773) (Rebagliati et al. 2003).

Studies on the characterization and localization of heterochromatin are important because in addition to the numerous functions that it performs during the cell cycle, it is related to karyotype evolution since chromosomal breaks and rearrangements occur frequently in these regions (Huisinga et al. 2006; Grewal and Jia 2007). The occurrence of small CMA_3_^+^ blocks and/or dots related to co-localization with the nucleolus-organizing regions is a common feature (Camacho et al. 1985; Rebagliati et al. 2001; Bardella et al. 2013b). We confirmed this for *E. collaris*, where the heterochromatic dots showed specificity to the fluorochrome CMA_3_, and subsequently by 18S rDNA hybridization. A higher percentage of CG repeats in the nucleotide composition of the 18S gene has already been observed by Bargues et al. (2000) in study with triatomines.

Signals of 18S rDNA in a single bivalent were observed for all species of this study, and this pattern is commonly found in the species of the Pentatomidae (Papeschi et al. 2003; Grozeva et al. 2015). Most studies of the Pentatomidae report the presence of this cluster on an autosomal pair (Papeschi et al. 2003; Cattani and Papeschi 2004; Cattani et al. 2004; Bressa et al. 2008, 2009; Grozeva et al. 2011, 2015; Bardella et al. 2013a, 2016a; Souza-Firmino et al. 2020).

In this study, first data on FISH with the 18S rDNA probe with *D. melacanthus* and *L. viridis* are presented and both species showed terminal blocks in autosomes, being the larger bivalent in *D. melacanthus*. This terminal location is highly conserved in the infraorder Pentatomomorpha, even in related species that exhibit wide variations in chromosome number; chromosome position of the 18S rDNA sites is commonly sub-terminal (Bardella et al. 2013a). The species *E. collaris* showed two signals of hybridization in an autosomal bivalent, as previously reported by Souza-Firmino et al. (2020).

Our results confirm the karyotype conservation of the family and present original cytogenetic data for three species: (i) analysis of heterochromatin in all species; and (ii) FISH with 18S rDNA probe data for *D. melacanthus* and *L. viridis*. In conclusion, we present new data for future studies that can collaborate in the evolutionary study of the Pentatomidae family.
